# Different Stages of Cardiovascular–Kidney–Metabolic Syndrome Associated With the Risk of All‐Cause and Cause‐Specific Mortality: Insights From Two Prospective Cohort Studies

**DOI:** 10.1155/ije/1409909

**Published:** 2026-08-02

**Authors:** Xiangyu Wang, Mengge Yang, Qunwei Ma, Xiaona Chang, Guang Wang, Jia Liu, Jian Wu

**Affiliations:** ^1^ College of Public Health, Zhengzhou University, Zhengzhou Henan, 450001, China, zzu.edu.cn; ^2^ Department of Endocrinology, Beijing Chao-Yang Hospital, Capital Medical University, Beijing 100020, China, ccmu.edu.cn

**Keywords:** cardiovascular disease, cardiovascular–kidney–metabolic syndrome, chronic kidney disease, mortality, stage

## Abstract

**Aims:**

Stage 3 in cardiovascular–kidney–metabolic (CKM) syndrome, defined as subclinical cardiovascular disease (CVD), diagnosis often relies on risk equivalents (very high‐risk CKD or high 10‐year CVD risk) due to the limited accessibility of diagnostic tools. A critical unanswered question is whether these two risk‐equivalent subgroups exhibit comparable mortality risks. This study compares mortality risks between these subgroups.

**Methods:**

The study included 344,515 participants from the UK Biobank and 24,545 participants from the National Health and Nutrition Examination Survey (NHANES). Participants were stratified according to AHA diagnostic criteria into Stage 0, 1, 2, 3, and 4. Stage 3 was further subclassified as 3a (10‐year high risk of CVD) and 3b (very high‐risk CKD). Cox proportional hazards regression models were applied to assess the risks of all‐cause, cardiovascular, renal, and cancer mortality across different stages.

**Results:**

In the UK Biobank, compared with Stage 0, Stage 3b was associated with the second‐highest multivariable‐adjusted hazard ratios (HRs) for all‐cause mortality (4.95; 95% CI: 4.41–5.55), cardiovascular mortality (18.75; 95% CI: 14.70–23.91), renal mortality (59.73; 95% CI: 21.53–165.68), and cancer mortality (2.88; 95% CI: 2.33–3.56), second only to Stage 4b. These risks were significantly higher than those observed in Stage 3a and even exceeded those in Stage 4a. The analysis of the NHANES cohort also validated the results for all‐cause mortality and cardiovascular mortality risks described above.

**Conclusion:**

The patients in Stage 3b with very high‐risk CKD, even in the absence of clinical CVD, face a very high risk of mortality and should be given significant attention and early intervention.

## 1. Introduction

Metabolic, cardiovascular, and kidney diseases frequently coexist, with growing evidence supporting the pathophysiological interactions between these conditions [1, 2]. The concept of cardiovascular–kidney–metabolic (CKM) syndrome was first defined by the American Heart Association (AHA), where it was outlined as a framework for assessing cardiovascular, kidney, and metabolic health [3]. It represents a state of health disorder characterized by these interconnected factors, encompassing individuals at high risk for cardiovascular disease (CVD) and those with established CVD, such as heart failure (HF), atrial fibrillation, coronary artery disease, stroke, and peripheral artery disease [3]. CKM syndrome contributes to multiorgan dysfunction and notably increases the risk of adverse outcomes [4–6]. Recent studies have indicated that approximately 80%–90% of adults are affected by CKM syndrome, with an increasing number of individuals progressing to the advanced stages (Stage 3 or 4) of CKM syndrome [7–9]. CKM syndrome poses a significant burden on global public health and the economy.

Current evidence suggests that CKM syndrome is a progressive condition characterized by excessive and dysfunctional adipose tissue, which leads to inflammation, oxidative stress, and insulin resistance and progresses to a series of metabolic risk factors, including hypertension, hypertriglyceridemia, metabolic syndrome (MetS), and Type 2 diabetes [10]. The AHA has also proposed a staging construct for CKM syndrome to reflect its progressive nature. Accurate staging is essential for reflecting the disease’s progression and plays a critical role in risk assessment, early implementation of preventive interventions, and prognosis improvement. However, whether the current staging system can accurately reflect patients’ prognosis and mortality risk remains an unresolved issue. In patients with Stage 3 CKM syndrome, defined as subclinical CVD, diagnosis often relies on risk equivalents due to the limited accessibility of diagnostic tools such as coronary catheterization, CT angiography, and cardiac biomarkers. These risk equivalents include very high‐risk chronic kidney disease (CKD) and a high predicted 10‐year risk of CVD. Whether these two risk‐equivalent subgroups exhibit comparable mortality risks has been unknown. Therefore, this study included 344,515 individuals from the UK Biobank and 24,545 individuals from the National Health and Nutrition Examination Survey (NHANES). The patients in Stage 3 were subcategorized into 3a (defined as the 10‐year high risk of CVD) and 3b (defined as very high‐risk CKD), and the relationship between different stages of CKM syndrome and the risks of all‐cause, cardiovascular, renal, and cancer mortality were assessed in the present study.

## 2. Methods

### 2.1. Study Subjects

UK Biobank is an ongoing population‐based cohort study involving over 500,000 community‐dwelling individuals aged 40–69 years from 22 assessment centers across England, Scotland, and Wales. Baseline assessments were conducted between 2006 and 2010. Ethical approval for the UK Biobank was granted by the North West Multi‐Centre Research Ethics Committee. Written informed consent was obtained from all participants. After excluding individuals lacking the key variables essential for the CKM syndrome definition, as well as the relevant covariates, a total of 344,515 participants from the UK Biobank were included in the study (Application No. 425612).

NHANES was designed to assess the health and nutritional status of the general US population. It combines interviews and physical examinations to collect data on a wide range of health‐related topics, including chronic conditions, dietary habits, physical fitness, and environmental factors. A stratified, multistage probability sampling method is used to select a series of nationally representative cross‐sectional samples. Since 1999–2000, the survey has been conducted in 2‐year cycles. For the current analysis, data from seven cycles spanning 2005–2006 to 2017–2018 were used. The study protocol was approved by the Institutional Review Board of the National Center for Health Statistics, and written informed consent was obtained from all participants. After excluding individuals lacking the key variables essential for the CKM syndrome definition, as well as the relevant covariates, a total of 24,545 participants from NHANES were included in the study.

The inclusion and exclusion criteria for the UK Biobank and NHANES cohorts in this study are detailed in Supporting Figure 1.

### 2.2. Assessment of CKM Syndrome Stages

The CKM syndrome classification system, as outlined in the AHA advisory, includes five stages, which in this study we refer to as the AHA‐defined CKM stage [2]. In this study, the stage definitions were adapted to align with the data available in different cohorts.

 Stage 0 comprised participants with a normal body mass index (BMI) (< 23 kg/m^2^ for individuals of Asian ethnicity and < 25 kg/m^2^ for other racial groups), normal waist circumference (< 80 cm for women and < 90 cm for men of Asian descent, and < 88 cm for women and < 102 cm for men of other racial groups), and who did not meet the criteria for higher stages.
 Stage 1 included participants with an elevated BMI (≥ 23 kg/m^2^ for individuals of Asian descent and ≥ 25 kg/m^2^ for others), an elevated waist circumference (≥ 80 cm for women and ≥ 90 cm for men of Asian descent, and ≥ 88 cm for women and ≥ 102 cm for men in other groups), or prediabetes (glycated hemoglobin [HbA1c] of 5.7% to < 6.5% or fasting blood glucose [FBG] of 100 to < 126 mg/dL).
 Stage 2 included participants with elevated fasting serum triglycerides (TG) (≥ 135 mg/dL), hypertension, diabetes, or MetS (defined by the presence of at least three of the following criteria: elevated waist circumference, low high‐density lipoprotein cholesterol [high‐density lipoprotein (HDL) < 40 mg/dL for men or < 50 mg/dL for women], fasting serum TG ≥ 150 mg/dL, elevated blood pressure [systolic blood pressure (SBP) ≥ 130 mmHg, diastolic blood pressure (DBP) ≥ 80 mmHg, and/or use of antihypertensive medications], or prediabetes), as well as moderate‐to‐high‐risk CKD per the Kidney Disease Improving Global Outcomes (KDIGO) criteria (https://kdigo.org/wp-content/uploads/2024/03/KDIGO-2024-CKD-Guideline.pdf).
 Stage 3 encompasses subclinical CVD or risk equivalents of subclinical CVD. Subclinical CVD is defined as either subclinical ASCVD or subclinical HF in individuals with excess or dysfunctional adiposity, other metabolic risk factors, or CKD. Subclinical ASCVD is primarily diagnosed through the presence of coronary artery calcification; alternative diagnostic methods, such as coronary catheterization or CT angiography that reveals subclinical atherosclerosis, also meet the diagnostic criteria. Subclinical HF is identified by elevated cardiac biomarkers, specifically NT‐proBNP levels of ≥ 125 pg/mL, high‐sensitivity troponin T levels of ≥ 14 ng/L for women and ≥ 22 ng/L for men, and high‐sensitivity troponin I levels of ≥ 10 ng/L for women and ≥ 12 ng/L for men. Risk equivalents of subclinical CVD included very high‐risk CKD per KDIGO criteria or high predicted 10‐year CVD risk. High predicted 10‐year CVD risk was defined as a predicted 10‐year CVD risk of ≥ 20%, as estimated using the AHA Predicting Risk of CVD EVENTs (PREVENT) equations, which included age, sex, total cholesterol (TC), HDL, SBP, BMI, estimated glomerular filtration rate (eGFR), diabetes status, smoking status, treatment for hypertension, and statin use [11]. Due to the lack of direct indicators, this study defines Stage 3 using risk equivalents of subclinical CVD. To assess the effectiveness of risk equivalents of subclinical CVD in evaluating mortality risk and to enable a more precise risk stratification of patients in Stage 3, our study further subcategorized this stage into 3a (defined as the 10‐year high risk of CVD) and 3b (defined as very high‐risk CKD) in the present study. Participants who met criteria for both Stage 3a and Stage 3b were classified into Stage 3b, given the higher mortality risk associated with this subgroup as demonstrated by our findings.
 Stage 4 included participants with clinical CVD (such as coronary heart disease, HF, stroke, peripheral artery disease, or atrial fibrillation) accompanied by excess or dysfunctional adiposity, other CKM syndrome risk factors, or CKD. Stage 4a: no kidney failure. Stage 4b: kidney failure present. Kidney failure was defined as having an eGFR < 15 mL/min/1.73 m^2^ or receiving kidney replacement therapy (dialysis or kidney transplantation), in accordance with the KDIGO clinical practice guidelines for CKD.

### 2.3. Variables Extraction

We extracted data on age, sex, BMI, race, education level, Townsend deprivation index (a higher score indicates worse socioeconomic status, UK Biobank only), HbA1c, HDL, TC, TG, FBG, alanine aminotransferase (ALT), aspartate aminotransferase (AST), serum creatinine, urine protein, urine creatinine, and C‐reactive protein (CRP) from participants in the UK Biobank and NHANES cohorts.

The UK Biobank cohort examined various health outcomes, including all‐cause, cardiovascular, renal, and cancer mortality. Death data were obtained from the death registry. All‐cause mortality was defined as death from any cause during the follow‐up period. Other outcomes were coded according to ICD‐10: cardiovascular mortality (Codes I00–I99), renal mortality (Codes N00–N39), and cancer mortality (Codes C00–C97). The follow‐up period was defined as the time from recruitment to either the date of death or the last follow‐up. The field IDs of all variables are listed in Supporting Table 1.

In NHANES, the vital status of participants was determined by a match between NHANES personal identifiers and connections to death certificates from the National Death Index until December 31, 2019. Cardiovascular mortality was identified using ICD‐10 (Codes I00–I09, I11, I13, and I20–I51). The follow‐up period was defined as the time from recruitment to either the date of death or the last follow‐up.

The eGFR was calculated using the 2021 Chronic Kidney Disease Epidemiology Collaboration (CKD‐EPI) formula [12]. The urinary albumin‐to‐creatinine ratio (UACR) was calculated by dividing the urinary albumin concentration (mg/dL) by the urinary creatinine concentration (g/dL). The smoking history, alcohol consumption history, use of antihypertensive medications, use of lipid‐lowering medications, and use of antidiabetic medications were all obtained through questionnaire surveys.

### 2.4. Statistical Analysis

Baseline participant characteristics were summarized as mean ± standard deviation (SD) or median (interquartile range) for continuous variables and as counts with percentages for categorical variables, stratified by CKM stages. The Kolmogorov–Smirnov test was used to evaluate normality. Clinical characteristics of patients across different CKM stages were compared using analysis of variance (ANOVA) for normally distributed data and the Kruskal–Wallis test for non‐normally distributed data. Categorical variables were compared using the chi‐square test. Cox proportional hazards regression models were applied to assess the risks of all‐cause, cardiovascular, renal, and cancer mortality across different CKM stages, with results expressed as hazard ratio (HR) and 95% confidence intervals (CIs). Three models were applied to investigate various mortality risks among different stages: Model 1, the unadjusted model, did not adjust for any covariates; Model 2 adjusted for age and sex; and Model 3 (the fully adjusted model) further adjusted for age, sex, race, education level, Townsend deprivation index (UK Biobank only), smoking status, alcohol consumption, AST, ALT, and CRP (UK Biobank only). Additionally, based on the complex survey design, the NHANES data were adjusted by incorporating stratification, primary sampling units, and probability weights into the statistical model using survey analysis procedures. All analyses were performed with R (Version 4.4.2). A two‐sided *p* < 0.05 was considered as statistically significant.

## 3. Results

### 3.1. Baseline Characteristics of the Participants According to Different Stages of CKM Syndrome

A total of 344,515 participants from the UK Biobank cohort were included in the study. The characteristics of participants according to different CKM stages are presented in Table 1. Among the 344,515 participants in the UK Biobank cohort, the mean age was 56.51 ± 8.11 years, and 47.06% were male. Compared to participants in Stage 0, those in Stage 3 and Stage 4, particularly Stage 3b and Stage 4b, exhibited the lowest socioeconomic status and educational attainment (all *p* < 0.05). They demonstrated significantly elevated levels of BMI, ALT, AST, HbA1c, TG, UACR, and CRP, alongside reduced levels of HDL and eGFR (all *p* < 0.05). Furthermore, these groups showed a higher prevalence of hypertension and diabetes (all *p* < 0.05).

**TABLE 1:** Baseline characteristics of participants according to CKM syndrome stages in the UK Biobank cohort.

Characteristics	Overall	Stage 0	Stage 1	Stage 2	Stage 3	Stage 4	*p*
No. of participants	344515.00	36179.00	48516.00	229844.00	5682.00	24294.00	
Age, years	56.51 (8.11)	51.59 (7.67)	53.49 (8.02)	57.23 (7.78)	64.60 (4.72)	61.15 (6.57)	< 0.001
Male, *n* (%)	162128 (47.06)	9860 (27.25)	16160 (33.31)	115750 (50.36)	4408 (77.58)	15950 (65.65)	< 0.001
BMI, kg/m^2^	27.53 (4.72)	22.38 (1.73)	27.43 (3.58)	28.11 (4.69)	30.20 (5.05)	29.26 (4.94)	< 0.001
Smoking status, *n* (%)							
Never	187044 (54.47)	22431 (62.12)	28261 (58.39)	124726 (54.45)	1891 (33.48)	9735 (40.30)	< 0.001
Previous	120114 (34.98)	10051 (27.84)	15423 (31.86)	81113 (35.41)	2145 (37.98)	11382 (47.11)	
Current	36208 (10.55)	3626 (10.04)	4719 (9.75)	23209 (10.13)	1612 (28.54)	3042 (12.59)	
Drinking status, *n* (%)							
Never	14960 (4.35)	1253 (3.47)	1945 (4.01)	10055 (4.38)	321 (5.66)	1386 (5.72)	< 0.001
Previous	12197 (3.54)	1053 (2.91)	1498 (3.09)	7738 (3.37)	331 (5.83)	1577 (6.50)	
Current	316947 (92.11)	33840 (93.62)	45016 (92.90)	211785 (92.25)	5023 (88.51)	21283 (87.78)	
Ethnicity, *n* (%)							
Non‐White	17759 (5.17)	1602 (4.44)	2993 (6.19)	11581 (5.06)	412 (7.28)	1171 (4.84)	< 0.001
White	325545 (94.83)	34484 (95.56)	45352 (93.81)	217440 (94.94)	5247 (92.72)	23022 (95.16)	
Townsend deprivation index	−1.34 (3.05)	−1.44 (2.99)	−1.35 (3.02)	−1.39 (3.02)	−0.93 (3.21)	−0.76 (3.32)	< 0.001
ALT, U/L	23.56 (13.99)	17.51 (9.42)	20.43 (12.16)	24.81 (14.36)	27.94 (17.16)	26.01 (15.13)	< 0.001
AST, U/L	26.16 (9.93)	23.79 (8.85)	24.54 (9.23)	26.61 (9.85)	28.37 (13.03)	28.18 (11.53)	< 0.001
HbA1c (%)	5.45 (0.61)	5.19 (0.28)	5.32 (0.34)	5.47 (0.59)	6.41 (1.22)	5.77 (0.90)	< 0.001
HDL (mg/dL)	55.54 (14.16)	65.09 (14.35)	59.96 (13.79)	54.04 (13.35)	44.70 (10.16)	49.12 (13.43)	< 0.001
TC (mg/dL)	219.06 (40.86)	211.11 (36.88)	215.53 (38.18)	224.97 (39.55)	201.20 (39.81)	186.25 (44.29)	< 0.001
TG (mg/dL)	155.29 (88.86)	85.06 (23.92)	94.51 (23.52)	176.14 (90.77)	219.52 (118.25)	168.96 (93.86)	< 0.001
CRP (mg/L)	2.61 (4.32)	1.36 (3.38)	2.37 (4.42)	2.77 (4.26)	3.92 (5.56)	3.16 (5.16)	< 0.001
eGFR, mL/(min × 1.73m^2^)	95.01 (13.03)	103.46 (6.90)	96.59 (11.72)	94.32 (12.68)	81.71 (21.93)	88.91 (15.32)	< 0.001
UACR (mg/g)	28.59 (144.65)	19.72 (71.34)	17.11 (56.51)	23.66 (95.39)	123.68 (515.08)	51.29 (225.20)	< 0.001
Diabetes, *n* (%)	24916 (7.23)	0 (0.00)	0 (0.00)	16285 (7.09)	4010 (70.57)	4621 (19.02)	< 0.001
Hypertension, *n* (%)	189895 (55.12)	0 (0.00)	0 (0.00)	167012 (72.66)	5534 (97.40)	17349 (71.41)	< 0.001

*Note:* Data are presented as mean ± standard deviation, or *n* (%). ALT, alanine aminotransferase; AST, aspartate aminotransferase; HbA1c, hemoglobin A1c; TG, triglycerides.

Abbreviations: BMI, body mass index; CKM, cardiovascular–kidney–metabolic; CRP, C‐reactive protein; eGFR, estimated glomerular filtration rate; HDL, high‐density lipoprotein; TC, total cholesterol; TDI, Townsend deprivation index; UACR, urinary albumin‐to‐creatinine ratio.

The validation cohort included a total of 24,545 participants from the NHANES study (Supporting Table 2). The weighted population was 148,479,151, with a weighted mean age of 50.92 ± 12.93 years, and 48.61% were males. Consistent with the findings from the UK Biobank cohort, the NHANES cohort also demonstrated similar distribution characteristics. Significant differences were observed across the different CKM stages. Generally, participants in more advanced CKM stages tended to be older and male, with higher levels of BMI, ALT, AST, HbA1c, FBG, TG, and UACR but lower levels of HDL and eGFR (all *p* < 0.001). Additionally, they exhibited a higher prevalence of hypertension and diabetes (all *p* < 0.001).

### 3.2. Association Between Different Stages of CKM Syndrome and the Risk of All‐Cause Mortality

**FIGURE 1:**
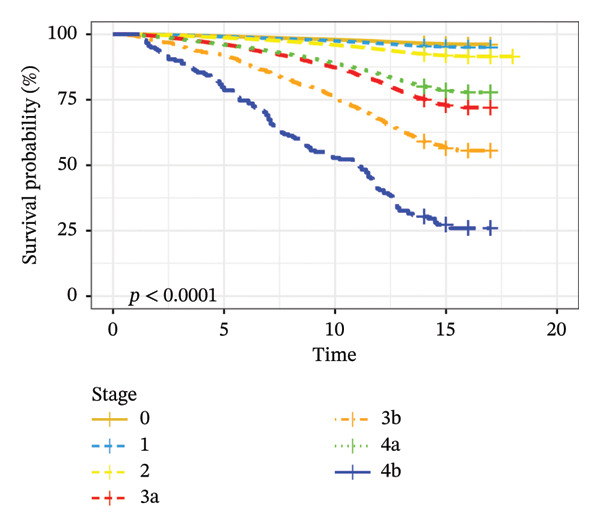
Kaplan–Meier analysis of survival probability stratified by CKM stage in the UK biobank cohort. Abbreviations: CKM, cardiovascular–kidney–metabolic.

**FIGURE 2:**
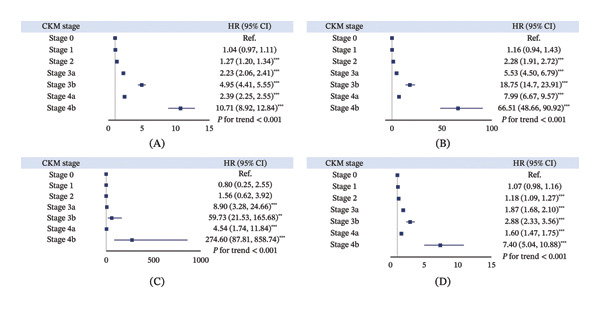
The various mortality risks stratified by CKM stage in the UK Biobank cohort. (A) All‐cause mortality; (B) cardiovascular mortality; (C) renal mortality; and (D) cancer mortality. Abbreviations: CKM, cardiovascular–kidney–metabolic; HR, hazard ratio; CI, confidence interval; Ref, reference (Stage 0, HR = 1). Model 1: unadjusted. Model 2: adjusted for age and sex. Model 3: Model 2 + race, education level, Townsend deprivation index, alanine aminotransferase, aspartate aminotransferase, and C‐reactive protein. ^∗^ *p* < 0.05; ^∗∗^ *p* < 0.01; ^∗∗∗^ *p* < 0.001.

In the UK Biobank cohort, the average follow‐up time was 14.82 ± 2.19 years, with a total of 29,398 deaths. The Kaplan–Meier survival curves demonstrated significant divergence, with Stage 4b exhibiting the lowest survival probability, followed by Stage 3b, Stage 3a, Stage 4a, Stage 2, Stage 1, and Stage 0 (*p* < 0.0001) (Figure 1). Additionally, multivariate Cox regression was performed to evaluate all‐cause mortality across different CKM syndrome stages (Table 2 and Figure 2A). A graded increase in mortality risk was observed with advancing stages of CKM syndrome (*p* for trend < 0.001). After adjusting for all covariates, compared to Stage 0, Stage 4b exhibited the highest adjusted HR (95% CI) of all‐cause mortality 10.71 (8.93, 12.84), followed by 4.95 (4.42, 5.55) for Stage 3b, 2.39 (2.25, 2.55) for Stage 4a, 2.23 (2.06, 2.41) for Stage 3a, and 1.27 (1.20, 1.34) for Stage 2 (all *p* < 0.001).

**TABLE 2:** The various mortality risks stratified by different CKM syndrome stage in the UK Biobank cohort.

		HR (95% CI)		
		Model 1	Model 2	Model 3
All‐cause mortality	0	Ref.	Ref.	Ref.
	1	1.29 (1.21, 1.38)^∗∗∗^	1.06 (0.99, 1.13)	1.04 (0.97, 1.11)
	2	2.25 (2.13, 2.37)^∗∗∗^	1.30 (1.22, 1.37)^∗∗∗^	1.27 (1.20, 1.34)^∗∗∗^
	3a	8.24 (7.65, 8.88)^∗∗∗^	2.43 (2.25, 2.63)^∗∗∗^	2.23 (2.06, 2.41)^∗∗∗^
	3b	15.04 (13.44, 16.84)^∗∗∗^	5.83 (5.21, 6.54)^∗∗∗^	4.95 (4.42, 5.55)^∗∗∗^
	4a	6.38 (6.01, 6.78)^∗∗∗^	2.62 (2.46, 2.79)^∗∗∗^	2.39 (2.25, 2.55)^∗∗∗^
	4b	34.65 (28.92, 41.51)^∗∗∗^	13.10 (10.92, 15.71)^∗∗∗^	10.71 (8.93, 12.84)^∗∗∗^

Cardiovascular mortality	0	Ref.	Ref.	Ref.
	1	1.46 (1.18, 1.80)^∗∗∗^	1.18 (0.96, 1.46)	1.16 (0.94, 1.43)
	2	4.25 (3.58, 5.06)^∗∗∗^	2.31 (1.94, 2.75)^∗∗∗^	2.27 (1.90, 2.70)^∗∗∗^
	3a	23.70 (19.43, 28.92)^∗∗∗^	6.03 (4.91, 7.39)^∗∗∗^	5.53 (4.50, 6.79)^∗∗∗^
	3b	61.63 (48.49, 78.34)^∗∗∗^	22.04 (17.29, 28.10)^∗∗∗^	18.75 (14.70, 23.91)^∗∗∗^
	4a	23.84 (20.00, 28.44)^∗∗∗^	8.80 (7.36, 10.54)^∗∗∗^	7.99 (6.67, 9.57)^∗∗∗^
	4b	220.36 (161.56, 300.54)^∗∗∗^	79.33 (58.05, 108.40)^∗∗∗^	66.51 (48.66, 90.92)^∗∗∗^

Renal mortality	0	Ref.	Ref.	Ref.
	1	1.06 (0.34, 3.33)	0.84 (0.26, 2.64)	0.78 (0.25, 2.48)
	2	2.97 (1.21, 7.31)^∗^	1.54 (0.62, 3.83)	1.49 (0.59, 3.73)
	3a	45.76 (17.52, 119.53)^∗∗∗^	10.69 (3.95, 28.90)^∗∗∗^	9.00 (3.28, 24.66)^∗∗∗^
	3b	243.00 (90.22, 654.51)^∗∗∗^	80.47 (29.28, 221.20)^∗∗∗^	59.73 (21.53, 165.68)^∗∗∗^
	4a	15.32 (6.06, 38.78)^∗∗∗^	5.39 (2.09, 13.90)^∗∗∗^	4.54 (1.74, 11.84)^∗∗^
	4b	1073.82 (351.28, 3282.56)^∗∗∗^	351.62 (112.88, 1095.23)^∗∗∗^	274.60 (87.81, 858.74)^∗∗∗^

Cancer mortality	0	Ref.	Ref.	Ref.
	1	1.33 (1.22, 1.45)^∗∗∗^	1.10 (1.01, 1.20)^∗^	1.07 (0.98, 1.16)
	2	2.05 (1.91, 2.21)^∗∗∗^	1.22 (1.13, 1.31)^∗∗∗^	1.18 (1.09, 1.27)^∗∗∗^
	3a	6.50 (5.84, 7.24)^∗∗∗^	2.06 (1.84, 2.30)^∗∗∗^	1.87 (1.68, 2.10)^∗∗∗^
	3b	8.03 (6.51, 9.91)^∗∗∗^	3.33 (2.69, 4.11)^∗∗∗^	2.88 (2.33, 3.56)^∗∗∗^
	4a	3.97 (3.65, 4.33)^∗∗∗^	1.74 (1.60, 1.90)^∗∗∗^	1.60 (1.47, 1.75)^∗∗∗^
	4b	21.22 (14.45, 31.14)^∗∗∗^	8.54 (5.81, 12.54)^∗∗∗^	7.40 (5.04, 10.88)^∗∗∗^

*Note:* Model 1: adjusted for age and sex. Model 2: Model 1 + race, education level, Townsend deprivation index, alanine aminotransferase, aspartate aminotransferase, and C‐reactive protein. Ref, reference (HR = 1).

Abbreviations: CI, confidence interval; CKM, cardiovascular–kidney–metabolic; HR, hazard ratio.

^∗^ *p* < 0.05.

^∗∗^ *p* < 0.01.

^∗∗∗^ *p* < 0.001.

**FIGURE 3:**
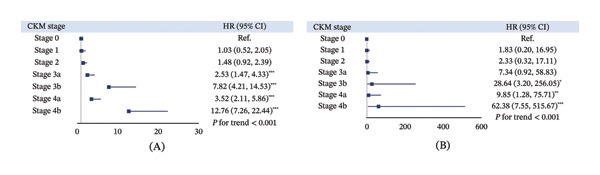
The various mortality risks stratified by CKM stage in the NHANES cohort. (A) All‐cause mortality and (B) cardiovascular mortality. Model 1: unadjusted. Model 2: adjusted for age and sex. Model 3: Model 2 + race, education level, Townsend deprivation index, alanine aminotransferase, aspartate aminotransferase, and C‐reactive protein. ^∗^ *p* < 0.05; ^∗∗^ *p* < 0.01; ^∗∗∗^ *p* < 0.001. Abbreviations: CKM, cardiovascular–kidney–metabolic; HR, hazard ratio; CI, confidence interval; Ref, reference (Stage 0, HR = 1).

In the NHANES cohort, the average follow‐up time was 7.61 ± 3.92 years, with a total of 2076 deaths. Consistent with the results from the UK Biobank cohort, the Kaplan–Meier survival curve demonstrated that Stage 4b exhibited the lowest survival probability, followed by Stage 3b, Stage 3a, Stage 4a, Stage 2, Stage 1, and Stage 0 (*p* < 0.0001) in the NHANES cohort (Supporting Figure 2). After adjusting for all covariates, compared to Stage 0, Stage 4b exhibited the highest adjusted HR (95% CI) of all‐cause mortality 12.76 (7.26, 22.44), followed by 7.82 (4.21, 14.53) for Stage 3b, 3.52 (2.11, 5.86) for Stage 4a, and 2.53 (1.47, 4.33) for Stage 3a (all *p* < 0.001) (Figure 3A).

### 3.3. The Association Between Different Stages of CKM Syndrome and the Risk of Cardiovascular, Renal, and Cancer Mortality

We also assessed the risks of cardiovascular, renal, and cancer mortality across different CKM syndrome stages. In the UK Biobank cohort, the risk of cardiovascular, renal, and cancer mortality increased progressively with advancing stages, regardless of covariate adjustment (*p* for trend < 0.001) (Table 2 and Figure 2B–D). After adjusting for all covariates, compared to Stage 0, Stage 4b exhibited the highest adjusted HR (95% CI) of cardiovascular mortality 66.51 (48.66, 90.92), followed by 18.75 (14.70, 23.91) for Stage 3b, 7.99 (6.67, 9.57) for Stage 4a, 5.53 (4.50, 6.79) for Stage 3a, and 2.27 (1.90, 2.70) for Stage 2 (all *p* < 0.001) (Figure 2B). Stage 4b also exhibited the highest adjusted HR (95% CI) of renal mortality 274.60 (87.81, 858.74) and cancer mortality 7.40 (5.04, 10.88), followed by Stage 3b, Stage 4a, Stage 3a, and Stage 2 (*p* for trend < 0.001) (Figure 2C, D).

Due to the limited data on renal and cancer mortality in the NHANES cohort, we used the NHANES data only to validate the differences in cardiovascular mortality risks associated with different CKM stages. Consistent with the findings from the UK Biobank cohort, the risk of cardiovascular mortality increased progressively with the advancement of CKM syndrome stages after adjusting for all covariates (*p* for trend < 0.001) (Figure 3B). Adjusting for all covariates, compared with Stage 0, Stage 4b exhibited the highest adjusted HR (95% CI) of cardiovascular mortality 62.38 (7.55, 515.67), followed by Stage 3b, Stage 4a, Stage 3a, and Stage 2 (*p* for trend < 0.001).

## 4. Discussion

This study evaluated the association between different stages of CKM syndrome and the risks of all‐cause mortality, as well as cardiovascular, renal, and cancer mortality. The findings from two cohort studies conducted in the UK Biobank and the NHANES consistently demonstrated that the various mortality risks progressively increased with the advancement of CKM syndrome stages, which was consistent with a Chinese study [13]. Notably, Stage 4b, characterized by the coexistence of clinical CVD and CKD, was associated with a significantly higher mortality risk compared to all other stages.

Several critical insights emerged from this investigation. First, although the overall mortality risk exhibited a progressive increase with the staging of CKM syndrome, significant heterogeneity in mortality risk was observed even within the same stage. According to the AHA presidential advisory, patients with high 10‐year CVD risk calculated by the PREVENT equation and those with very high‐risk CKD according to the KDIGO staging criteria are typically categorized as subclinical CVD (Stage 3). To more accurately assess the mortality risk in these patients, we further subdivided them into Stage 3a (high 10‐year CVD risk) and Stage 3b (very high‐risk CKD). Our findings revealed that patients in Stage 3b exhibited significantly elevated risks of all‐cause mortality, as well as cardiovascular, renal, and cancer‐related mortality. Notably, their mortality risk was second only to that of Stage 4b patients and even surpassed that of Stage 4a patients, who have clinical CVD.

Both previous research and our study suggest that CKD should be given more attention as contributors to mortality [2, 14, 15]. CKD has been established as an independent risk factor for cardiovascular mortality, and patients with very high‐risk CKD often present with advanced renal dysfunction, predisposing them to a substantially higher risk of developing CVD. Once cardiovascular events occur in this population, they tend to progress rapidly, significantly amplifying mortality risk [16–19]. Furthermore, severe renal impairment greatly increases the likelihood of developing other comorbidities, such as cerebrovascular events, gastrointestinal bleeding, and other systemic complications [20–24]. These additional health risks further exacerbate the progression and contribute to an elevated risk of all‐cause mortality in this population. The presence of severe kidney dysfunction can lead to a cascading effect on multiple organ systems, resulting in a much higher disease burden and a compounded mortality risk. These findings underscore that CKD should not be regarded merely as a risk factor for CVD and emphasize the necessity of early intervention and comprehensive management strategies for patients with very high‐risk CKD.

Additionally, we observed that patients in Stage 3a exhibited higher risks of renal and cancer mortality compared to those in Stage 4a. Stage 3a encompassed individuals with high 10‐year cardiovascular risk, who were assessed using the PREVENT formula, which integrates various factors, including sex, age, TC, high‐density lipoprotein, SBP, diabetes mellitus, smoking, BMI, eGFR, blood pressure treatment, and statin use [11]. These patients with high 10‐year cardiovascular risk typically had higher baseline age and BMI, along with poorer liver and kidney function, as well as worse blood pressure and blood glucose control [13, 25]. Although patients in Stage 3a have not yet developed clinical CVD, they exhibited significantly worse metabolic conditions. This metabolic dysfunction may explain why they had a higher risk of renal and cancer mortality, which suggests that metabolic status plays a crucial role in CKM syndrome prognosis. This observation aligns with existing literature, which indicates that metabolic abnormalities are significant risk factors for both kidney disease and cancer mortality [26–30].

These findings provide important insights: When staging and managing CKM syndrome patients, adequate attention should be given even to those who have not yet progressed to clinical CVD. Clinicians should prioritize aggressive screening for CKD in all CKM syndrome patients, especially those with metabolic risk factors, even in the absence of overt CVD symptoms. This implies a need for earlier and more intensive interventions targeting kidney health, such as optimized blood pressure and glucose control, appropriate nephroprotective medications (e.g., SGLT2 inhibitors and GLP‐1 receptor agonists), and close monitoring of renal function, even before overt cardiovascular events occur. Recognizing Stage 3b as a distinct, exceptionally high‐risk category could lead to a paradigm shift in CKM syndrome management, advocating for proactive nephrological care similar to, or even exceeding, the urgency given to early CVD management.

To our knowledge, this represents the first multinational investigation utilizing both UK Biobank and NHANES cohorts to systematically examine associations between CKM syndrome and all‐cause, cardiovascular, renal, and cancer mortality. However, several limitations need to be addressed. Firstly, biomarker measurements were not fully available; therefore, we were unable to analyze patients with subclinical HF in Stage 3. Secondly, while the UK Biobank provided robust population‐level data, analytical power for renal and cancer mortality endpoints was substantially constrained by the NHANES cohort’s limited sample size; thus, these were not validated in NHANES. Additionally, for cardiovascular mortality in Stage 4b within the NHANES cohort, the very wide CI suggests a small number of events and less stable estimates, a limitation inherent to studies with smaller event counts in specific subgroups. Although the datasets used are broadly representative, future studies should prioritize validating these findings in more diverse cohorts, particularly those encompassing varied racial and geographical populations, to enhance the generalizability and robustness of the results.

## 5. Conclusion

The mortality risks progressively increase with the advancement of CKM syndrome stages, particularly in those with concurrent clinical CVD and CKD. Notably, even among patients with very high‐risk CKD who have not yet developed clinical CVD, their mortality risk remains exceedingly elevated. Consequently, this patient population requires paramount attention in clinical practice. Early identification and proactive intervention should be emphasized to effectively reduce their mortality risk.

## Author Contributions

M.Y., X.W., and J.W. designed the study. J.L. and G.W. collected the data. J.L. conducted the data analysis. M.Y. and X.W. wrote the manuscript. X.C., M.Y., and Q.M. supervised and revised the manuscript.

## Funding

This work was supported by grants from the National Key R&D Program of China (2022YFA0806401) and National Natural Science Foundation (82470889) to Guang Wang and National Natural Science Foundation (82570988) and High Innovation Program (G202522117) to Jia Liu.

## Disclosure

All the authors have read and approved the final manuscript.

## Ethics Statement

The UK Biobank was approved by the North West Multi‐Centre Research Ethics Committee (16/NW/0274). This study was performed under UK Biobank application number 425612. The NHANES study protocol was approved by the NCHS Research Ethics Review Board.

## Consent

All UK Biobank participants provided written and informed consent for data collection, analysis, and record linkage. Written informed consent was provided by all NHANES participants.

## Conflicts of Interest

The authors declare no conflicts of interest.

## Supporting Information

Additional supporting information can be found online in the Supporting Information section.

## Supporting information


**Supporting Information** Supporting Table 1. The field IDs of all variables in UKB cohort. Supporting Table 2. Baseline characteristics of the study population stratified by CKM Stage in the NHANES cohort. Data are presented as mean ± standard deviation, or n (%). Abbreviations: CKM, cardiovascular‐kidney‐metabolic; BMI, body mass index; ALT, alanine aminotransferase; AST, aspartate aminotransferase; TC, total C=cholesterol; TG, triglycerides; HbA1c, hemoglobin A1c; HDL, high‐density lipoprotein; LDL, low‐density lipoprotein; eGFR, estimated glomerular filtration rate; UACR, urinary albumin‐to‐creatinine ratio. Supporting Figure 1. Flowchart of the study population. Abbreviations: NHANES, National Health and Nutrition Examination Survey; BMI, body mass index; FBG, fasting blood glucose; SBP, systolic blood pressure; DBP, diastolic blood pressure; TG, triglycerides; TC, total cholesterol. Supporting Figure 2. Kaplan–Meier analysis of survival probability stratified by CKM Stage in NHANES cohort. Abbreviation: CKM, cardiovascular‐kidney‐metabolic.

## Data Availability

Data from the UK Biobank cannot be shared publicly. However, data are available from the UK Biobank Institutional Data Access/Ethics Committee (contact: https://www.ukbiobank.ac.uk) for researchers who meet the criteria for access to the confidential data. The data from the National Health and Nutrition Examination Survey are openly available at https://www.cdc.gov/nchs/about/index.html.
