# Supplementary material for: Different Stages of Cardiovascular–Kidney–Metabolic Syndrome Associated With the Risk of All‐Cause and Cause‐Specific Mortality: Insights From Two Prospective Cohort Studies
Source: Int J Endocrinol. 2026 Aug 2;2026:1409909. doi: 10.1155/ije/1409909 (PMC13430281; doi:10.1155/ije/1409909)
Supplement: Supplementary file 1 — Supporting Information Supporting Table 1. The field IDs of all variables in UKB cohort. Supporting Table 2. Baseline characteristics of the study population stratified by CKM Stage in the NHANES cohort. Data are presented as mean ± standard deviation, or n (%). Abbreviations: CKM, cardiovascular‐kidney‐metabolic; BMI, body mass index; ALT, alanine aminotransferase; AST, aspartate aminotransferase; TC, total C=cholesterol; TG, triglycerides; HbA1c, hemoglobin A1c; HDL, high‐density lipoprotein; LDL, low‐density lipoprotein; eGFR, estimated glomerular filtration rate; UACR, urinary albumin‐to‐creatinine ratio. Supporting Figure 1. Flowchart of the study population. Abbreviations: NHANES, National Health and Nutrition Examination Survey; BMI, body mass index; FBG, fasting blood glucose; SBP, systolic blood pressure; DBP, diastolic blood pressure; TG, triglycerides; TC, total cholesterol. Supporting Figure 2. Kaplan–Meier analysis of survival probability stratified by CKM Stage in NHANES cohort. Abbreviation: CKM, cardiovascular‐kidney‐metabolic. [file IJE-2026-1409909-s001.docx]

**Supplement Table 1. The field IDs of all variables in UKB cohort.**

| **Field ID** | **Variable** |
| --- | --- |
| 21003 | Age |
| 31 | Sex |
| 48 | Waist circumferenc |
| 49 | Hip circumference |
| 4079 | Diastolic blood pressure |
| 4080 | Systolic blood pressure |
| 30750 | Glycated haemoglobin |
| 30740 | Glucose |
| 30650 | Aspartate aminotransferase |
| 30620 | Alanine aminotransferase |
| 30690 | Cholesterol |
| 30760 | HDL cholesterol |
| 30780 | LDL direct |
| 30870 | Triglycerides |
| 30790 | Lipoprotein A |
| 30700 | Creatinine |
| 30510 | Creatinin |
| 30500 | Microalbumin in urine |
| 30710 | C-reactive protein |
| 30880 | Urate |
| 6177 | Medication for cholesterol, blood pressure or diabetes |
| 6153 | Medication for cholesterol, blood pressure, diabetes, or take exogenous hormones |
| 21000 | Ethnic background |
| 22189 | Townsend deprivation index at recruitment |
| 20117 | Alcohol drinker status |
| 20116 | Smoking status |
| 738 | Average total household income before tax |
| 6138 | Education |
| 41270 | Diagnoses - ICD10 |
| 41280 | Date of first in-patient diagnosis - ICD10 |
| 53 | Date of attending assessment centre |
| 191 | Date lost to follow-up |
| 40000 | Date of death Death |

**Supplement Table 2. Baseline characteristics of the study population stratified by CKM Stage in the NHANES cohort**

| **Characteristic** | Overall | **CKM syndrome Stage** | | | | | | | P |
| --- | --- | --- | --- | --- | --- | --- | --- | --- | --- |
|  |  | 0 | 1 | 2 | 3a | 3b | 4a | 4b |  |
| **Weighted number** | 148,479,151 | 9,858,787 | 12,347,545 | 107,291,412 | 6,090,352 | 890,786 | 11,440,198 | 560,071 |  |
| **Age, years** | 50.92 (12.93) | 42.55 (9.82) | 43.24 (10.29) | 50.06 (11.78) | 71.71 (6.79) | 64.12 (12.29) | 61.60 (11.35) | 67.72 (9.26) | <0.001 |
| **Male, n (%)** | 11,963 (48.61%) | 522 (36.28%) | 705 (43.10%) | 8,367 (49.17%) | 896 (57.64%) | 116 (42.67%) | 1,272 (55.94%) | 85 (41.32%) | <0.001 |
| **BMI, kg/m2** | 29.56 (6.75) | 22.00 (1.95) | 29.41 (4.74) | 29.97 (6.72) | 30.22 (6.43) | 32.88 (9.40) | 31.70 (7.23) | 31.92 (7.42) | <0.001 |
| **Race** |  |  |  |  |  |  |  |  | <0.001 |
| Mexican American | 3,983 (8.06%) | 167 (6.21%) | 393 (11.66%) | 2,905 (8.29%) | 204 (5.68%) | 38 (9.16%) | 262 (4.92%) | 14 (5.78%) |  |
| Other Hispanic | 2,507 (5.35%) | 146 (6.05%) | 231 (7.34%) | 1,775 (5.31%) | 130 (4.03%) | 17 (5.03%) | 197 (3.90%) | 11 (2.76%) |  |
| Non-Hispanic White | 10,218 (68.97%) | 626 (70.48%) | 691 (65.06%) | 6,984 (68.86%) | 676 (73.62%) | 77 (52.41%) | 1,105 (72.30%) | 59 (56.80%) |  |
| Non-Hispanic Black | 5,064 (10.09%) | 150 (5.27%) | 331 (9.72%) | 3,541 (10.12%) | 355 (11.43%) | 95 (25.85%) | 517 (11.39%) | 75 (29.56%) |  |
| Other Race | 2,773 (7.53%) | 336 (11.99%) | 161 (6.22%) | 1,961 (7.42%) | 106 (5.24%) | 20 (7.55%) | 178 (7.49%) | 11 (5.10%) |  |
| **College graduate or above** | 5,928 (31.31%) | 573 (47.66%) | 550 (39.25%) | 4,187 (31.07%) | 250 (20.11%) | 34 (14.49%) | 318 (19.14%) | 16 (9.90%) | <0.001 |
| **ALT (U/L)** | 21.00 (17.00, 29.00) | 18.00 (14.00, 22.00) | 20.00 (16.00, 27.00) | 22.00 (17.00, 30.00) | 20.00 (16.00, 26.00) | 17.00 (13.00, 22.00) | 21.00 (17.00, 28.00) | 18.00 (14.00, 21.00) | <0.001 |
| **AST (U/L)** | 23.00 (19.00, 27.00) | 22.00 (19.00, 26.00) | 22.00 (18.00, 26.00) | 23.00 (19.00, 28.00) | 24.00 (20.00, 29.00) | 21.00 (17.00, 24.00) | 23.00 (19.00, 28.00) | 21.00 (17.00, 26.00) | <0.001 |
| **TC (mg/dL)** | 200.28 (37.14) | 190.10 (31.22) | 194.33 (30.78) | 203.91 (37.34) | 194.16 (37.10) | 189.17 (45.22) | 186.42 (38.76) | 182.84 (43.16) | <0.001 |
| **Glucose (mg/dL)** | 101.46 (35.31) | 87.42 (9.98) | 90.35 (9.76) | 100.78 (34.04) | 125.57 (52.91) | 130.26 (79.60) | 115.08 (44.59) | 137.88 (68.18) | <0.001 |
| **TG (mg/dL)** | 161.54 (119.74) | 76.68 (26.18) | 85.26 (26.10) | 174.26 (124.51) | 190.77 (128.02) | 188.95 (132.28) | 178.46 (116.43) | 195.84 (133.57) | <0.001 |
| **HbA1c, %** | 5.68 (0.95) | 5.26 (0.31) | 5.31 (0.34) | 5.66 (0.92) | 6.39 (1.27) | 6.69 (1.58) | 6.16 (1.24) | 6.58 (1.39) | <0.001 |
| **HDL, mg/dL** | 53.16 (15.13) | 65.18 (13.01) | 60.06 (12.18) | 51.95 (15.01) | 48.90 (13.72) | 50.56 (13.71) | 49.33 (14.50) | 48.97 (15.21) | <0.001 |
| **LDL, mg/dL** | 118.84 (32.54) | 110.13 (30.02) | 119.34 (28.83) | 121.93 (32.60) | 109.23 (31.98) | 101.41 (36.83) | 106.71 (33.06) | 101.33 (34.63) | <0.001 |
| **eGFR,**  **ml/（min×1.73m^2^）** | 93.14 (18.66) | 106.75 (9.62) | 106.08 (9.70) | 93.44 (16.87) | 73.68 (18.04) | 33.32 (14.49) | 82.70 (18.78) | 31.30 (13.35) | <0.001 |
| **UACR, mg/g** | 30.78 (244.11) | 6.60 (4.44, 11.62) | 6.20 (4.49, 9.09) | 5.13 (3.84, 7.59) | 6.46 (4.36, 11.20) | 12.18 (7.00, 27.31) | 195.65 (48.39, 1,260.76) | 8.89 (5.42, 19.05) | <0.001 |
| **Hypertension, n(%)** | 14,411 (54.92%) | 0 (0.00%) | 0 (0.00%) | 10,730 (60.66%) | 1,389 (94.38%) | 232 (93.17%) | 1,897 (81.68%) | 163 (96.85%) | <0.001 |
| **Diabetes, n(%)** | 4,941 (15.30%) | 0 (0.00%) | 0 (0.00%) | 2,880 (13.29%) | 891 (56.91%) | 149 (58.93%) | 911 (35.74%) | 110 (67.07%) | <0.001 |

Data are presented as mean ± standard deviation, or n (%).

Abbreviations: CKM, cardiovascular-kidney-metabolic; BMI, body mass index; ALT, Alanine Aminotransferase; AST, Aspartate Aminotransferase; TC, Total Cholesterol; TG, triglycerides; HbA1c, Hemoglobin A1c; HDL, high-density lipoprotein; LDL, low-density lipoprotein; eGFR, estimated glomerular filtration rate; UACR, urinary albumin-to-creatinine ratio.


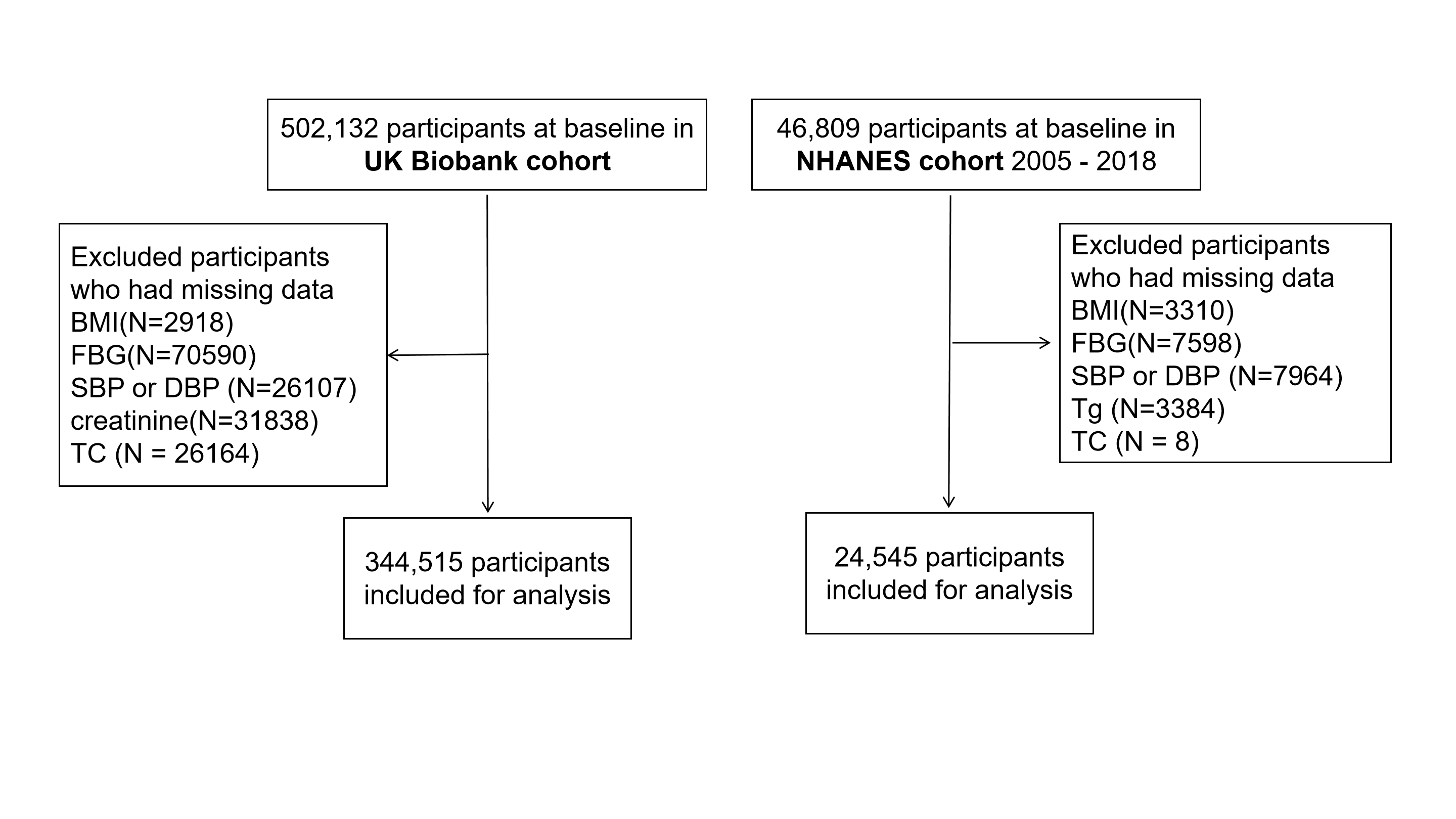


**Supplement Figure 1.  Flowchart of the study population.**

Abbreviations: NHANES, National Health and Nutrition Examination Survey; BMI, body mass index; FBG, fasting blood glucose; SBP, systolic blood pressure; DBP, diastolic blood pressure; TG, triglycerides; TC, total cholesterol


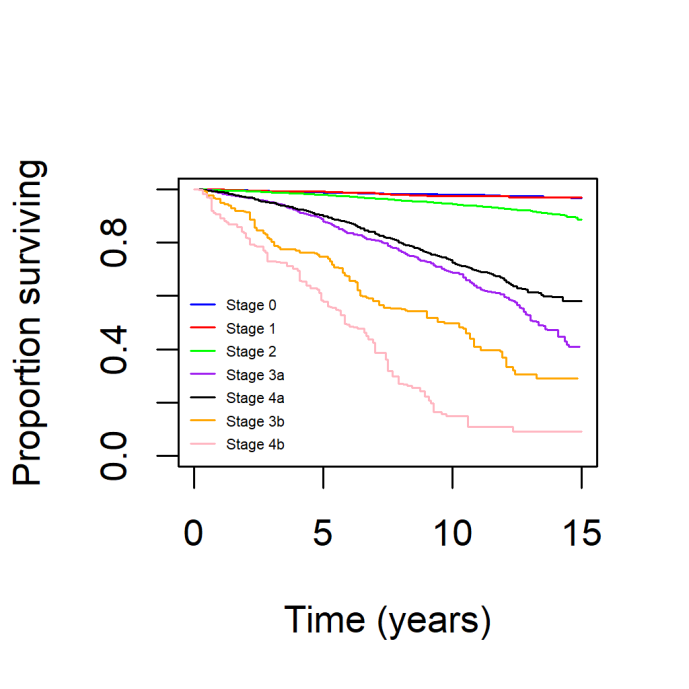


**Supplement Figure 2. Kaplan-Meier analysis of survival probability stratified by CKM Stage in NHANES cohort**

Abbreviations: CKM, cardiovascular-kidney-metabolic
